# Incorporating equity in economic evaluations: a multi-attribute equity state approach

**DOI:** 10.1007/s10198-017-0897-3

**Published:** 2017-06-01

**Authors:** Jeff Round, Mike Paulden

**Affiliations:** 1 School of Social and Community Medicine, University of Bristol, Canynge Hall, 39 Whatley Road, Bristol, BS8 2PS UK; 2 School of Public Health, University of Alberta, Edmonton, Canada

**Keywords:** Equity, Equity weights, Distributional weights, Cost-effectiveness, I1 Health, I10 General, I14 Health and Inequality, I18 Government Policy, Regulation, Public Health, I19 Other

## Abstract

In publicly funded health care systems, decision makers must continually balance often conflicting priorities of efficiency and equity. Health economists have developed a set of highly sophisticated analytical methods for assessing efficiency, but less attention has been paid to formally incorporating equity considerations into analyses. As a result, where equity is considered is often informal, ad hoc and/or simplistic. This paper is a proposal for a mechanism for formally incorporating equity within the decision process. It begins with an overview of the current literature on equity weighting. It then considers the case of a single equity domain and illustrates how this is currently applied in practice by the UK’s National Institute for Health and Care Excellence. It then proposes a more comprehensive method for considering the multi-attribute equity state, where a population exhibits more than one trait considered worthy of differential weighting. Finally, the paper proposes a mechanism by which this could be applied in practice, and concludes with a discussion of the challenges for applying multi-attribute equity weighting.

**Electronic supplementary material:**

The online version of this article (doi:10.1007/s10198-017-0897-3) contains supplementary material, which is available to authorized users.

## Introduction

Those tasked with allocating scarce health care resources in publicly funded health care systems are required to balance often conflicting aims of efficiency and equity. Health economists have developed a set of highly sophisticated analytical methods for assessing efficiency, but less attention has been paid to formally incorporating equity considerations into analyses. As a result, where equity is considered, the process is often informal, ad hoc, simplistic and lacking in transparency. The most commonly recommended approach for considering equity in the decision making process is the application of an equity (or distributional) weight [1, 2], though to date no decision making bodies have formally adopted such an explicit approach.

Yet some decision making bodies acknowledge that they do think about weighting criteria, even if this is not typically done explicitly or through formal methods. While occasionally equity weighting is explicit in individual decision processes, it is often implicit and revealed only through the decisions themselves [3]. And while many countries make reference to equity related issues in the technical guidance used to guide decision makers [4], these are frequently specified in terms of deliberative processes for considering equity concerns or relate to consideration of clinical sub-groups. But, as has been argued elsewhere [5], the complexity of considering equity in a deliberative process is staggering. To do so in a transparent way, that meets the standards required of procedural justice, is nigh on impossible. A current challenge for the research community is therefore how (or even whether) QALYs should be weighted across different categories of individuals in order to address concerns of equity in resource allocation decisions.

There are numerous difficulties that arise in trying to estimate and apply equity weights, and Wailoo et al. [6] highlight many of these. Perhaps the most challenging problem is that patient populations, even when sharing the same illness, are heterogeneous in many other respects, and are therefore likely to be possessed of many different attributes that might be considered worthy of equity weighting. To deal with the challenges of the population that exhibits multiple equity relevant attributes, this paper draws on recent literature to propose a formal mechanism for incorporating multiple equity-related attributes within the decision process—the multi-attribute equity state (MAES).

The paper begins with an overview of the current literature on equity weighting. Consideration is then given to the simple case of a single equity domain in order to illustrate how equity weights are currently applied in practice by the UK’s National Institute for Health and Care Excellence (NICE). A proposal is then outlined for a more comprehensive formal method for considering equity weighting in populations that exhibit more than one trait considered worthy of differential weighting. The paper concludes with a discussion of the challenges for applying multi-attribute equity weighting. It is not the aim of this paper to consider the arguments about whether ‘a QALY is a QALY’—this has been done elsewhere (for some examples of this literature, see McCabe et al. [5], Round [7], Donaldson et al. [8] and Dolan et al. [9]). Irrespective of the view one takes on the principle of differential weighting of QALYs, in practice it is becoming more common—in the UK alone, differential weights have been applied to treatments for those at the end of life [10], for those with cancer [11], for childhood vaccines [12], and for very rare diseases [13]. Rather, this paper is concerned with principles of procedural justice—if differential weighting is to be undertaken, it should be done in a manner that is fair, transparent, and has democratic legitimacy.

## Equity weights in economic evaluation

For many, the notion that ‘a QALY is a QALY’ is a fundamental principle of resource allocation decision making and cost-utility analysis [7, 14]. While this principle is widely assumed in the practice of economic evaluation, it is not universally accepted. It has been suggested by some that there are occasions when differential consideration should be given to health gains (or other benefits of treatment) based on the characteristics of those receiving care. It has been argued, for example, that the principle of equal value for all QALY gains is disadvantageous to people at the end of life [15, 16], that it is ageist [17, 18] and that it is based on assumptions about the characteristics of individuals that do not hold [19–21]. As a result, a literature has developed on how it might be possible to give greater weight, and therefore greater access to scarce resources, to some populations over others. Such weights are variously referred to as distributional weights or equity weights, referring to the idea that, by weighting health gain, a more equitable distribution of health can be achieved relative to the dominant health maximisation approach implied in the acceptance of the idea that ‘a QALY is a QALY’.

### An overview of the current literature

Much of the literature on equity weighting is concerned with the identification of attributes for weighting or estimating weights for individual attributes. A review from Paulden et al. [22] identified 19 individual candidate attributes for weighting from the existing literature. And in recent years, a number of empirical studies on equity weights and distributional concerns have been published. A recent systematic review [2] identified 64 such studies, published between 1989 and 2014. All studies included in that review reported on the identification of attributes deemed to be important in weighting, from a range of different populations (most commonly the UK, US and Australia). Studies included in the review were mixed as to whether they focused on a single attribute (28 studies) or multiple attributes (36 studies). A minority (22 studies) also attempted to estimate distributional weights for the identified attributes. Of these, 19 studies estimated weights for single attributes, and three studies [23–25] that estimated weights for multiple attributes (and of these, two are based on the same research into the social value of the QALY [23, 24]). Research that focuses on the existence of preferences between characteristics is also ongoing [26].

The Social Value of a QALY (SVQ) research project [23, 24] estimated weights using two different methods—a discrete choice experiment (DCE) and a person trade-off exercise (PTO). This research found that irrespective of the method by which weights were derived, people expressed a preference for health gains that are accrued by young people or the elderly. Only one attribute apart from age was considered to be important in health distribution; using the PTO method (but not in the DCE), it was found that illness severity should be given consideration.

The other study identified by Gu et al. [2] to estimate multi-attribute weights is from Dolan and Tsuchiya [25], who examined the trade-off between maximising total health gain against reducing inequalities in health. In this study, weights were estimated through trying to estimate a social-welfare function, incorporating equity considerations. They showed that a sample of the general public gave greater weight to health gain when a person was expected to die at the age of 60 compared to someone expected to die at the age of 70. They also found that the less time a person spent in full health, the greater the weight the respondents gave to gains in health. The overall results from this study suggest that age at death, and the length of time lived in good health, are both important priorities for equity weighting among the general public.

The most recent study to consider equity weighting is from Rowen and colleagues [3], and was undertaken as part of a programme of work looking at value-based pricing in decision making [27]. This study focused on whether greater value should be placed on populations according to the burden of illness they experience (which might otherwise be described as severity) and/or whether they are end-of-life. The results of this work found that both the ‘severity’ and ‘end-of-life’ were considered important considerations for weighting in decision making. This study was limited by design to considering burden of illness and end-of-life as potential equity weights.

The SVQ work represents the most comprehensive effort to date to understand the effects of multiple equity attributes on public preferences for health gain. While this work did identify a small number of instances where health gains would be differently valued by the public, most of the estimated weights were small [23]. By contrast, Dolan and Tsuchiya found relatively large weights applied to differences in the age of the beneficiary at onset and at death [25]. One possible reason for the discrepancy between the two studies is the number of attributes that respondents were asked to consider. In the SVQ project, respondents were asked to consider attributes relating to age at death, age at onset of ill-health, the severity of health lost and the potential health gain from treatment. Dolan and Tsuchiya [25] only required respondents to consider the age at death and age of onset of ill-health. This supports the idea that there are important interactions to be considered in developing equity weights.

Another possible source of difference in the two sets of results is the approach used to estimate weights. Dolan and Tsuchiya [25] used an approach based on trying to identify a latent social welfare function based on the stated preferences of individuals. The SVQ project team undertook a discrete choice experiment [23] and person-trade-off exercise [24]. Given differences in the underlying conceptual basis of each method, it is possible that this would lead to different weights being obtained. If the difference in results is down to the empirical method selected, then the wide variation in estimated weights should raise concerns about the validity of any results obtained to date. Further research into the most appropriate methods of deriving weights should be considered a priority.

### Equity weighting in current practice

As highlighted above, although much research has been done to derive individual or joint weights for equity attributes, there is no accepted formal system for applying weights in economic evaluations. Yet, despite this, weighting is routinely done, both implicitly [28] and explicitly [12], in the practice of decision making. This creates obvious problems. Such an approach lacks transparency, leads to a simplistic consideration of equity and is likely to result in a series of inconsistent, ad hoc decisions. The end-of-life criteria as applied by NICE are an ideal example of the flawed current process.

**Table:** Box 1. NICE end-of-life criteria

The treatment is indicated for patients with a short life expectancy, normally less than 24 months and;	
There is sufficient evidence to indicate that the treatment offers an extension to life, normally of at least an additional 3 months, compared to current NHS treatment, and;	
The treatment is licensed or otherwise indicated, for small patient populations	

In 2009, NICE introduced a series of criteria that would apply to cost-effectiveness decision making processes for a small subset of treatments, in a subset of patients (see Box 1). The rationale for this was that this patient group was somehow disadvantaged by the existing systems and did not receive an equitable allocation of resources. The practical effect of this policy has been to introduce a distributional weight for allocating resources when considering a particular population. Under the revised scheme, treatments meeting the new end-of-life criteria could be approved for use in the NHS under a less stringent threshold for cost-effectiveness than required for other treatments. Now, instead of being required to provide an additional QALY at a cost of £20,000 to £30,000, qualifying treatments must only generate each additional QALY at an incremental cost estimated to be in the region of £50,000 [29].

In effect this policy has created the first explicit equity weight to be used in practice by NICE—if the end-of-life threshold is in fact £50,000/QALY, then it is 2.5 times that of the lower limit of the standard threshold. If the threshold is taken to represent the opportunity cost of health displaced, this gives some sense of how much more weight decision makers place on the QALYs that accrue to the beneficiaries of the policy.

The approach chosen by NICE in relation to end-of-life treatments cannot be justified by equity concerns. Paulden and colleagues [30] have shown that simply applying a differential threshold to treatments or populations fails to identify who bears the opportunity cost of the additional weight given to the beneficiaries’ health. Where the patients who bear the opportunity cost of the decision are similar to those who gain, this can lead to differential weights being applied to similar patients, a violation of the principle of horizontal equity. The argument of Paulden et al. [30] is summarised as follows.

Consider a new treatment for a specific illness, where the opportunity cost is borne solely by other patients who also have that illness. Suppose that, for every £20,000 spent on the new treatment, one QALY is displaced among those patients who bear the opportunity cost. A healthcare payer wishes to assign 2.5× the value to QALYs for patients with the illness in question, and so decides to assign an acceptable threshold of £50,000 per QALY (2.5× the standard threshold of £20,000 per QALY). However, treatments approved at this threshold would displace 2.5 QALYs among patients who bear the opportunity cost—who in this example all have the same illness as the beneficiaries of the new treatment. The payer would be choosing to displace 2.5 QALYs in order to fund a treatment that generates just 1 QALY among patients with an identical illness. In this case, it would be more equitable to retain the original threshold. The revised threshold is only suitable where those who bear the opportunity cost have no overlap with the beneficiaries of treatment. Of course, in practice it might be unlikely that all patients who bear the opportunity cost have an identical illness to the beneficiaries—more likely, we would expect that the group of patients that bears the opportunity cost contains some with the illness in question (who should receive the same special consideration as the beneficiaries of the new treatment) and other patients who do not. This implies that the correct threshold should be somewhere between £20,000 and £50,000, depending upon the prevalence of the illness in question among the patients who bear the opportunity cost. This prevalence would only be revealed if the make-up of the opportunity cost group is known. For a full discussion see Paulden et al. [30].

In addition, the introduction of this equity weighting system has been undertaken with little regard to the methodological literature on applying equity criteria to economic evaluations. The Gu et al. review [2] identified seven studies that considered whether people value end-of-life differently in terms of distributional effects. Five of these studies found little to no evidence that this was the case. And among those studies that did find end-of-life to be an important criterion, only one attempted to estimate a distributional weight [31]. Pinto-Prades et al. estimate that the societal value of a QALY for a person at the end of life is 1.41 times greater than for others [31]. In the UK context and using a baseline threshold of £20,000/QALY, this would imply a threshold of £28,200 per QALY, lower even than the upper limit of the standard threshold, suggesting no need for specific criteria.

### Multi-attribute equity considerations

As illustrated in the Gu et al. review [2], it is feasible and relatively straightforward to estimate equity weights for single attributes. However, it is unrealistic to believe that those concerned with equity in provision of treatments are concerned only with single-attribute populations. It is far more likely that decision makers will be routinely concerned with multi-attribute populations in allocation decisions. For example, it is common for public discourse to suggest that both children and those at the end of life deserve exceptional consideration [12, 28], a clear example of a multi-attribute scenario.

It is also straightforward to apply single attribute weights in practice (assuming that, unlike the end-of-life criteria, they are evidence based). One possibility is that the weight could simply be applied directly to the QALY estimates used in the calculation of the cost-effectiveness ratio, thereby changing the ICER. Another approach, as taken by NICE, would be to apply a differential threshold criterion [10]—though it should be noted that these approaches may lead to different decisions and so should not be considered equivalent. For example, an intervention that is ‘dominated’ (i.e. more expensive and less effective than a comparator) without a weight applied may no longer appear dominated (and may even appear cost-effective) when the weight is applied. If, instead, the threshold is altered as an alternative to applying weights, then the dominated intervention will always appear dominated and so cannot appear cost-effective, irrespective of where the threshold is set [30]. It should also be borne in mind that while the end-of-life criteria may be weighted positively, weights may also be negative, reflecting attributes disfavoured by the public.

It is more difficult to deal with the multi-attribute scenario, and there are important methodological issues raised in a situation where there is a conjoint distributional problem. The most immediate solution to the problem would be to apply each weight individually in sequence. This approach works if it is assumed that weights are independent from one another and can be combined multiplicatively. Distributional weights estimated for individual attributes could then be applied, with no limit on the number of attributes considered. For the end-of-life child, the calculation is simply:

$$\frac{{\text{Incremental cost}}}{{\text{ (Incremental QALY } \times \text{ Weight}_{{\text{End of Life}}} \text{ } \times \text{ Weight}_{{\text{Child}}} \text{)}}}$$

It is unlikely, however, that equity weights associated with individual attributes are independent of one another. What is more likely is that the jointly estimated distributional weight (hereafter the joint-weight) is different from the product of the independently estimated weights. There is no conceptual basis on which to predict whether the joint-weight applied to the end-of-life child is greater than, less than, or equal to the product of the independently estimated weights. This can only be determined empirically. The Gu et al. review [2] highlighted the need for additional research on dealing with this joint distributional problem. In the UK, NICE proposed a maximum threshold of £50,000 per QALY when considering joint weights, although, as Paulden et al. show, this could lead to logically inconsistent decision making [30].

## Multi-attribute equity states

**Fig. 1:**
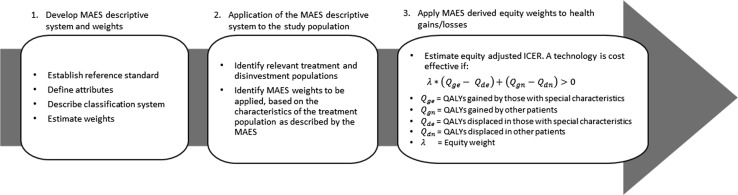
Proposed operational model of the multi-attribute equity state (MAES) approach

The following is a proposed solution to the challenge of incorporating multiple equity concerns within the decision making framework—the multi-attribute equity state (MAES). The framework for this system is guided by the recommendations of McCabe et al. [5]. It is illustrated in brief in Fig. 1. Nominally in reference to NICE’s end-of-life premium, McCabe et al. identify a set of criteria that any system that applies differential value to health gains must satisfy in order to be considered equitable. These are summarised in Table 1. As will be demonstrated, the proposed MAES framework satisfies the McCabe et al. criteria. In some respects, the proposed MAES is not new—a similar concept has been applied elsewhere in practice [24], though that discussion did not formalise the criteria for developing a comprehensive and consistent system that could be applied in a decision making context. It also did not address all of the criteria later set out by McCabe et al. [5].

**Table 1:** McCabe et al. criteria for equity weighting schemes

Criteria	Description
1	No single attribute should attract a value premium in isolation
2	Effects on those who bear the opportunity cost must be explicitly considered, not just benefits to the identified populations
3	Definitions for individual criteria must be validated against societal preferences
4	Weights must be applied equally to benefits in both quality and quantity of life
5	Weights must be empirically derived

The MAES framework is broadly analogous to the multi-attribute health state (MAHS) approach, as used to estimate utility values. Each approach has a reference value, is defined by a series of attributes and levels, and leads to the estimation of a weight applied to the health of the beneficiary of the health gain within the decision making process. In this section, further details are provided on the conceptual framework of the MAES, with particular reference to the MAHS classification approaches. The framework is also related throughout to the McCabe et al. [5] criteria described above. The next section discusses the MAES as it might be applied in practice.

### The reference standard

When estimating health state utility values the reference standard is full health—this is ascribed a maximum value of 1, on a scale typically anchored at 0 (representing the state of being dead). In the standard model, health states are then defined by a multi-attribute classification system, such as the EQ-5D [32]. Once defined, these states are then weighted according to a perceived deviation from the state of being in full health. In the EQ-5D classification and weighting system, the state defined by 11,111 is weighted as 1, meaning a year spent in that state is a year spent in full health. The state 22,222 describes a state of reduced health in all domains and is given a weight of 0.516 according to the UK EQ-5D-3L tariff [33], meaning a year lived in that state is the equivalent of roughly just half a year in full health.

To apply a system of multi-attribute equity states, a similar reference standard is needed. Unlike with health states, where the reference standard is full health, there is no obvious candidate for what the reference equity standard should be. One possibility is to anchor the system at 1 and 0, as with health states. In this approach, a weight of 1 applies where the population has a set of characteristics such that the net effect of these on the value of additional QALYs cancels out entirely (i.e. the same value is placed on additional QALYs for such individuals whether or not the additional weights are applied). A weight of 0 then applies when, given their characteristics, society places no value on additional QALYs for a patient or population. The system is bound by 0 at the lower end, but has no maximum positive weight. The proposal here is that the reference standard for a MAES is the case where the individual or population does not possess any particular characteristics considered to be deemed worthy of consideration for equity adjustments.

### Defining attributes

Defining the reference standard will require considerable investigation as to which attributes were deemed important to a population. This could be done through reference to the existing literature, as in the review from Paulden et al. [22] or through public surveys or focus groups, as per Baker et al. [24]. Public policy documents might also reveal attributes that are deemed relevant by decision makers. However, to maintain public support and to meet the demands of procedural justice (criterion 3, Table 1), any system for identifying equity attributes must at some stage be subject to public debate, be it through a process such as the NICE Citizen’s Council or through extensive empirical work and qualitative research with the general public. In addition, it must be recognised that the preference for attributes may change over time, as population norms and preferences change, and that this may require periodic updating of the MAES descriptive system and subsequently the MAES valuation set.

There are immediate difficulties apparent in trying to select attributes. Everyone has a race, sex and age, so defining which of these, if any, becomes part of the reference case is not straightforward. Should we include race/ethnicity as part of the reference case? Some illnesses are more prevalent in certain ethnic groups than in others and so this may be relevant. What about sex? And if we include sex, how should we consider inter-sexed people, or those who identify as a gender other than one assigned at birth? Would including these characteristics work to decrease disparities in health outcomes accordingly? We must also ask if the inclusion of certain characteristics would be in contravention of local or regional laws or institutional policies. In the UK, NICE is guided in incorporating equity considerations by both its own Social Values Judgment policy as well as anti-discrimination legislation [34]. Application of a MAES that violated these existing criteria could lead to a violation of procedural justice, and could be illegal.

Defining the reference group is clearly difficult, though it should be possible. However, it cannot be done in a single essay and no particular classification system is proposed here, rather a principle by which to establish one in future. To determine the reference case will require considerable research effort and public debate. A multi-attribute reference case derived from public preferences satisfies the first and third criteria identified in Table 1.

### Equity state classification system

The first step to determining the classification system is to determine what attributes it should contain, as discussed above. Once this has been done, it then must be decided how these attributes should be described within the classification system, similar to the way different levels of health attributes in MAHS classification system are described. For example, if age is selected as an attribute, it is possible to define it in the classification system in a multitude of ways. We may for instance include children, working age adults and adults older than the retirement age. Or we may class age in relation to the age at the onset of an illness. Or we may class age according to multiple such criteria. As with the attributes themselves, selecting the set of attributes must be done with reference to societal preferences, as any system of equity weighting that does not receive public support will be open to challenge on issues of procedural justice.

Once the attributes and levels have been selected, these combine to create a classification system, similar to a multi-attribute health state classification system. The classification system describes the complete set of equity-states that will then be used in the formal process of equity weighting. One possible approach for developing the overall classification system is for wider public consultation on the attributes of interest, with the operationalising of the attributes in the form of the classification system left to the research and policy community. Turning broad concepts such as age or illness severity into operational constructs is a complex and challenging endeavour requiring specialist skills. However, to maintain public input, the final classification system could be presented to the public for further consultation and refinement. This approach has been used in the development of MAHS classification systems [35].

### Weighting (or valuing) the MAES

Given an equity state classification system, it then becomes possible to rank and weight each state. Ranking can be undertaken using similar principles to the ranking of health states. Defining the equity state classification system defines the reference case and it is assigned a weight of 1.0. Other equity states are ranked and weighted relative to the reference case. The theoretical minimum value for an equity weight is 0, meaning that no resources would be allocated to that population despite any potential health gains. There is no maximum theoretical value. Equity weights can then be determined by public assessment of the various combinations of the possible equity states using choice based methods.

Previous attempts to estimate multi-attribute weights have used discrete choice experiments [3, 23], person-trade-off methods [24] and a social welfare function approach [25]. The most common approach to estimating weights for single attributes as described in the Gu et al. [2] review was the person-trade-off method. The results of the different attempts to estimate multi-attribute weights suggest that the method employed can lead to differing results, though this is confounded by the fact that each study was estimating weights for slightly different sets of attributes, classified in slightly different ways from one another. It is not clear from the small number of studies estimating weights for multiple attributes which method is best; in fact, it is not even clear how to define ‘best’.

It is important therefore that a set of conditions to determine which valuation method is most appropriate must be established a priori. As part of the SVQ project, Baker et al. undertook both a PTO and DCE, and each gave different results [24]. They concluded that “…the extent to which either approach yields results that are entirely consistent with social preferences is uncertain.” (Baker et al., p. 68, [36]). Not establishing a priori the grounds for choosing between different approaches risks, like Baker et al. [24], finding a range of different outcomes across different methods, with no means of choosing between them. It may be that multiple valuation methods are equally justifiable. If this is the case, clear decision criteria for choosing between the results of all methods should be stated at the outset of the study. In future, researchers may wish to register their research protocols with online repositories to illustrate what they plan to do ahead of doing it, reducing the risk of results being questioned later.

The criteria outlined below are necessary conditions for valuation studies of equity states:

1. The method must be choice based or ranking based. Respondents must be required to evaluate different alternatives and express preferences for options across the whole set of alternatives.
2. The task must not be cognitively demanding. Respondents must be able to understand easily what they are being asked to do. Cognitively demanding tasks risk respondents providing unreliable answers or not completing the whole task [24].
3. The task must be feasible. Some methods may require the valuation of large numbers of states, leading to non-completion by participants. Larger sample sizes valuing smaller numbers of states may facilitate this, but will then likely require online completion. A method that requires an unrealistic sample size will not be suitable.
4. The method and sample size must be appropriate for dealing with interactions between individual attributes.

Given the current state of the art, the most likely candidates to meet the above criteria are best-worst scaling approaches and discrete choice experiments. If a decision is made to undertake two different valuation exercises (for example, see Baker et al. [24] or Coast et al. [37]) there should be established conditions for choosing between each method. In choosing between two sets of results, one might consider whether one method generates a more consistent set of estimates [37], the reliability of the method (if the same experiment is run in a new population, are the results consistent?) or whether one method gives nonsensical results (e.g. equity weights of less than 0). Ranking and choice based methods satisfy the McCabe et al. criterion five, as listed in Table 1.

### Applying the MAES

Once the equity states have been ranked and valued, the weights can be applied in decision making. One obvious approach to doing so is to apply the weights to the utility values estimated for the patient population and used to calculate QALYs. If the population being considered is the reference case, then the utility value is simply multiplied by 1—effectively no equity weighting is applied. If the population differs in some characteristic from the reference population then the relevant equity weight is applied according to the ranking of that state. The process for this is simple—one just looks up the value corresponding to the equity state and applies that to the utility score. This adjusted utility value is then used in the QALY calculation in the standard way. This satisfies the 4th McCabe et al. criterion: that weights apply to both quality of life and length of life.

The most challenging component of this process is outlined by McCabe et al. [5] and concerns the role of the population from whom disinvestment in health is being made to fund the new treatment. Any allocation of resources results in an opportunity cost—those resources cannot be used to treat anyone else. When allocating resources, we typically know the identity of those receiving the treatment under consideration. However, we do not typically know the identities of those patients for whom treatment must be withdrawn. In the case where ‘a QALY is a QALY’ across all populations, then accounting for the opportunity cost simply requires estimating the health forgone, regardless of the identity and characteristics of those patients affected. A cost per QALY threshold, such as that estimated by Claxton et al. [38], is all that decision makers need to consider. However, if a QALY is not always QALY, then a cost per QALY threshold is insufficient. We need to know not only how many QALYs are displaced, but also the characteristics of those patients who lose QALYs, so we can apply weights to these QALYs accordingly.

#### Calculating gains and losses

To estimate the gains and losses we need to consider the patients to whom those gains and losses apply. We identify four distinct sets of QALY gains and losses to consider:

- $Q_{ge}$ = QALYs gained by those with special characteristics
- $Q_{gn}$ = QALYs gained by other patients (those with no special characteristics)
- $Q_{de}$ = QALYs displaced in those with special characteristics
- $Q_{dn}$ = QALYs displaced in other patients

The total QALYs gained are:

1 $$Q_{g} = Q_{ge} + Q_{gn}$$

The total QALYs displaced are:

2 $$Q_{d} = Q_{de} + Q_{dn}$$

In the case where a ‘QALY = QALY’, then a technology is cost-effective if:

3 $$Q_{g} > Q_{d}$$

In the weighted QALY calculation, if an intervention is favoured only after weighting, then by definition the unweighted QALYs gained will be less than the unweighted QALYs displaced. In the scenario where the populations gaining and losing QALYs are entirely distinct from one another according to the MAES, then the calculation is simple, applying equity weight *λ* to Eq. (3)

:

4 $$Q_{d} < Q_{g} \times \lambda$$

But this only necessarily holds if the disinvestment population is distinctly different from the intervention population across all equity criteria in the MAES. If the group from whom QALYs are disinvested also includes individuals who have the equity attribute(s) under consideration, this changes the number of unweighted QALYs required in the equity-favoured group in order for the intervention to be considered cost-effective. Where *λ* is applied to the QALYs of patients with special characteristics only, then the weighted QALYs gained are:

5 $$\hat{Q}_{g} = \left( {Q_{ge} \times \lambda } \right) + Q_{gn}$$

The weighted QALYs displaced are:

6 $$\hat{Q}_{d} = \left( {Q_{de} \times \lambda } \right) + Q_{dn}$$

And a technology is cost-effective if:

7 $$\hat{Q}_{g} > \hat{Q}_{d}$$

Alternatively, this could be expressed as:

8 $$\lambda *\left( {Q_{ge} - Q_{de} } \right) + \left( {Q_{gn} - Q_{dn} } \right) > 0$$

Identifying the proportion of people that may be in both the equity-favoured group and the displaced-care group may be difficult (and in some cases not possible). We typically do not know in a standard cost-effective analysis which groups stand to lose care as a result of an allocation decision, irrespective of weighting. The addition of an equity weight does not necessarily change this. If anything, it makes it more difficult—with equity weighting it will be necessary to identify exact sub-groups (and their size) for whom care is displaced in order to estimate $\hat{Q}_{d}$. This does not mean an equity weighting system should not be considered, but that the burden on analysts to identify those who bear the opportunity cost may be significant. An example of how the MAES would be applied in practice is provided in the online supplementary material.

### Can the MAES work in practice?

A system of equity weighting based on MAES, as outlined above, can work in principle. Conceptually, it is straightforward. It would require significant development and empirical work, but this would be little different, in principle, to the work that has underpinned the development of the cost-utility framework widely used in practice today. In addition, unlike the approaches applied by NICE in the end-of-life criteria [10], or the Joint Committee on Vaccination and Immunisation [12], the MAES can, in principle, satisfy the McCabe et al. criteria while not violating norms of procedural justice or horizontal equity.

The means of undertaking such research are becoming more accessible to researchers. While a MAES might represent a more technically complex problem than health state valuations in terms of ranking and weighting tasks, choice-based experimental methods are continuously being developed and refined, providing tools unavailable to those who first developed utility weights. It is not impossible to imagine a MAES tariff similar to that of the EQ-5D being developed. The SVQ project was such an attempt, and provides valuable insights in how to undertake further work. In addition, online survey technology is such that large samples of the population can be accessed to undertake DCEs or ‘best-worst scaling’ experiments. While there are arguments about the validity of online samples, steps can be taken to minimise the risks of bias [1]. In any case, any such risks must be weighed against the enormous cost in time and money that would be required to achieve an adequate sample size in face-to-face interviews. It is legitimate to ask whether an imperfect evidence base applied transparently is better than current systems, where little empirical evidence is applied and decisions are made deliberatively.

The construction of the MAES descriptive system, and the ranking and valuation of the resulting states, will present challenges to researchers. But these will not be the most difficult problem in the implementation of an equity weighting system. The greatest challenge will be estimating the values to assign to estimate the total burden of health displaced owing to weighting. Without including these values in the overall estimate, it is not possible to fairly assess the impact of weighting on those who bear the opportunity cost. But, as has been described above, it is not usually possible to identify specific groups from whom care will be withdrawn in order to invest elsewhere. Finding a solution to this particular challenge seems to be the most pressing research question relating to equity weighting. A system that applies equity weights to one population without accounting for those who lose out will not be fair or transparent, and seems unlikely to be able to maintain public favour over time.

#### Equity: horizontal or vertical?

That a QALY is a QALY is a specific vertical equity position, and one with which many are uncomfortable. There are also many reasonable alternative vertical equity positions where a QALY is not always a QALY. However, regardless of the vertical equity position adopted, it remains important to respect the principle of horizontal equity—treating individuals with similar characteristics in a similar way. Applying ‘equity’ weights only to the (identifiable) beneficiaries of a treatment, and not to the (similar but unidentifiable) bearers of the opportunity cost, violates this principle of horizontal equity [39]. This leaves the options of

1. Retaining the status quo, with all QALYs being equal, which maintains horizontal equity (by definition) but adopts a contentious vertical equity position, or
2. Moving to an alternative (and arguably more acceptable) vertical equity position but applying it in such a way that violates horizontal equity. The challenge here is to identify what level of horizontal inequity is acceptable, and justifying where the burden of that inequity should fall.

In an ideal world, we would satisfy both horizontal and vertical equity in a framework where a QALY is not always a QALY, but as identified above, this requires in-depth knowledge of the characteristics of the bearers of the opportunity cost (which might differ for each intervention and may not be possible to estimate) and a willingness to apply weights consistently across both the ‘winners’ and ‘losers’ such that horizontal equity is maintained, regardless of the vertical equity position adopted.

## Conclusion

Whether or not one agrees that equity weighting is appropriate in allocation decisions, it happens in practice and this is unlikely to change. It is therefore essential that equity concerns are incorporated into the decision making process in a way that is transparent, fair and conforms to standards of procedural justice. In the UK, the present system resoundingly fails to meet such standards—neither the end-of-life criteria nor the Cancer Drugs Fund meet a single criterion as set out by McCabe et al. [5]. The legitimacy of a system by which certain populations are favoured over others in resource allocation decisions rests solely on its acceptance to the public, for which the perception of transparency and fairness will be critical. The development of a conceptual framework for the incorporation of equity weighting into cost-utility analysis, as described above, is an important component of achieving these aims of transparency and fairness.

## Electronic supplementary material

Below is the link to the electronic supplementary material.

Supplementary material 1 (DOCX 32 kb)
