# Supplementary material for: Incorporating equity in economic evaluations: a multi-attribute equity state approach
Source: Eur J Health Econ. 2017 Jun 1;19(4):489–98. doi: 10.1007/s10198-017-0897-3 (PMC5913380; doi:10.1007/s10198-017-0897-3)
Supplement: Supplementary file 1 — Supplementary material 1 (DOCX 32 kb) [file 10198_2017_897_MOESM1_ESM.docx]

# A method for incorporating equity in economic evaluations: the multi-attribute equity state approach: Online supplementary material

## An illustration of the MAES as potentially applied in practice.

In this online supplementary material, we presented a worked example of how the MAES could be applied in practice. We begin by illustrating a sample MAES descriptive system and equity weight schedule of tariffs. We then describe a scenario under which the MAES would be relevant in a decision making context. Finally, we undertake the necessary calculations to illustrate the difference between the equity weighted decision and the non-equity weighted decision.

### A hypothetical MAES descriptive system

The 1^st^ stage in the development of the MAES is to define the descriptive system. The descriptive system includes both the high level equity domains, as well as the operational categories within each domain. In this example, we consider just two equity domains – age and illness severity [1]. We operationalise this as in Table S1. This results in an MAES defining a total of 20 equity states.

##### Table S1: A hypothetical MAES descriptive system

| Age | Illness severity |
| --- | --- |
| - Infancy (0-3) | - Healthy |
| - Childhood (4-16) | - Mild |
| - Working age (16-64) | - Moderate |
| - Retirement age (65 – 75) | - Severe |
| - Old age (76 and older) |  |

### The MAES tariff

For the purposes of the example, we consider the following MAES tariff of joint distributions for equity states. In this example a healthy person of working age, has an equity weight of 1.0. The remainder of the values are assigned to be consistent with the following principles:

- Infants and children are preferred to all adults
- The oldest adults are preferred to other adults
- More severe disease is weighted more highly than less severe disease

##### Table S2: A hypothetical MAES tariff of equity weights

| Illness severity | Healthy | Mild | Moderate | Severe |
| --- | --- | --- | --- | --- |
| Age |  |  |  |  |
| Infancy (0-3) | 1.30 | 1.35 | 1.45 | 1.80 |
| Childhood (4-15) | 1.20 | 1.22 | 1.30 | 1.60 |
| Working age (16-64) | 1.00 | 1.01 | 1.15 | 1.30 |
| Retirement age (65 – 75) | 1.05 | 1.05 | 1.15 | 1.25 |
| Old age (76 and older) | 1.20 | 1.30 | 1.40 | 1.50 |

### A hypothetical scenario

Consider the following scenario. A new treatment has been developed for children aged 10 – 15 with a severe illness. It is the only treatment available, and the alternative is to do nothing. There are 100 people to whom the treatment applies. Pre-treatment QALYs are 0.4 and post-treatment QALYs are 0.6, for an incremental gain of 0.2 QALYs per person, or 20 total QALYs. The incremental treatment costs are £8,000 per patient, giving an incremental cost effectiveness ratio (ICER) of £40,000 per QALY. To estimate the weighted QALY gains and losses we need to consider the patients to whom those gains and losses apply. From the main body of the paper recall that:

| $Q_{ge}$ | → QALYs gained by those with special characteristics → | Children (4-16), severe illness |
| --- | --- | --- |

For every QALY gained by $Q_{ge}$ we apply a weight of 1.60. So now the QALYs gained are equal to the unweighted QALY gain (20) multiplied by the weight (1.60), giving a weighted QALY gain of 32 (and a mean gain of 0.32). This gives an ICER of £25,000 per QALY, making the intervention more likely to be considered cost-effective. It also requires displacement of 32 QALYs from elsewhere in the health system, whereas the unweighted assessment required a displacement of just 20 QALYs. .

As discussed in the main body of the paper, this calculation only applies if the disinvestment population is distinctly different from the intervention population across all equity criteria in the MAES, such that no weight is applied to their QALYs. If the group from whom QALYs are disinvested also includes individuals who have the equity attribute(s) under consideration, this changes the number of unweighted QALYs required in the equity-favoured group in order for the intervention to be considered cost-effective.

Consider the alternative version of the above. The intervention is now only available to children under 15 years of age, but the illness affects individuals into young adulthood. In this case, the intervention will displace QALYs from an affected patient group (those with severe disease who are of working age) but who cannot benefit from the intervention. This effect is introduced solely due to the application of the equity weight.

In this case, imagine 20% of the disinvestment population of 35 working age adults have the illness, and 80% are healthy. The 28 healthy individuals have a mean QALY of 1.0, giving 28 total QALYs. The 7 patients with severe disease have a mean QALY of 0.4 and do not benefit from treatment, giving 2.8 total unweighted QALYs.

Applying *λ* to the QALYs of patients with special characteristics, then the mean weighted QALYs gained are as above:

$\hat{Q}_{g}= \left( Q_{ge}* \lambda\right)+Q_{gn} = \left( 20*1.60 \right)+0=32$

We now also consider the mean weighted QALYs displaced. These are equal to the proportion of people in the disinvestment group with the overlapping characteristic, as well as those from whom health is displaced but is unweighted. Recall again from the main body of the paper that QALYs displaced are:

$\hat{Q}_{d} =\left( Q_{de}* \lambda\right)+Q_{dn}$

Giving:

$\hat{Q}_{d} =\left( Q_{de}* \lambda\right)+Q_{dn} = \left( 0.20*35*0.4* 1.6 \right)+28 =4.48+28=32.48$

A technology will be cost-effective if, all other things being equal, the weighted QALY gain from the investment group is greater than the QALYs displaced in the disinvestment group, or $\hat{Q}_{g}>\hat{Q}_{d}$. From the above we can see that $\hat{Q}_{g}=32$ QALYs, and $\hat{Q}_{d}=32.48$ QALYs. In this scenario, the intervention would displace more QALYs than it would generate after applying the equity weight. The intervention will need to generate more health gain to be cost effective.

Supplementary references

1. Lancsar, E., et al., *Deriving distributional weights for QALYs through discrete choice experiments.* Journal of Health Economics, 2011. **30**(2): p. 466-478.
